# Abbreviating the Early Start Denver Model for community-based family-centered care

**DOI:** 10.3389/fpsyg.2023.1167885

**Published:** 2023-07-20

**Authors:** Laurie A. Vismara, Lucy Nyugen, Carolyn E. B. McCormick

**Affiliations:** ^1^ESDM Online, Toronto, ON, Canada; ^2^Thrive Autism Collaborative, Denver, CO, United States; ^3^Department of Human Development and Family Science, Purdue University, West Lafayette, IN, United States

**Keywords:** autism, community-based, early intervention (EI), family-centered care, telehealth

## Abstract

**Introduction:**

Parent-mediated approaches for young children with or with a higher likelihood of autism have gained traction, with mounting evidence of efficacy, but a research-to-gap practice exists, and community effectiveness remains to be firmly established.

**Methods:**

Using a community-participatory framework, a total of 10 parent-child dyads received a five-day workshop and six follow-up sessions of ESDM parent coaching. Intervention was implemented across two phases with in-person and telehealth delivery.

**Results:**

From pre to post intervention across both phases, parents improved in their fidelity of intervention implementation and children maid gains on proximal measures of social communication.

**Discussion:**

Community delivery of an evidence-based parent-mediated interventions for toddlers on the autism spectrum is feasible and promising. Giving resource efficiencies associated with parent-mediated approaches, particularly when delivered through government-funded programs, findings bolster current efforts to promote earlier and more widespread community access to necessary interventions. Facilitators and barriers to supporting parent learning and behavior change via interactive strategies are discussed.

## 1. Introduction

Specific interventions for young children with or with a higher likelihood of autism spectrum disorders (on the autism spectrum) demonstrate powerful effects in reducing intellectual impairment, improving social communication and language development, as well as social skills, when begun in the toddler period (e.g., Wallace and Rogers, 2010; Dawson et al., 2012; Schertz et al., 2013; Zwaigenbaum et al., 2015). Interventions that include parents as the agent of delivery have been shown to be particularly effective (Hampton and Kaiser, 2016) with long-term outcomes heavily reliant on parent participation (Kim et al., 2018). Parent efficacy and empowerment seem to increase with the more strategies they have to support their interactions and relationship with their child (Hume et al., 2005; Ingersoll and Dvortcsak, 2006; Bryson et al., 2007; Brian et al., 2017; Tomeny et al., 2020) to the point that early interventions (EI) services through Part C, the federally mandated program within the Individuals with Disabilities Education Improvement Act (2004, p. 108–446), endorse family-centered care as the best practice approach to service delivery.

In contrast to professional-centered approaches, family-centered care is characterized by a collaborative partnership between families and providers and considers the needs and priorities of the whole family. Family-centered care employs practices, such as shared decision-making, listening carefully, being sensitive to family values and customs, considering the psychosocial needs of all family members, and making caregivers feel like an equal partner (Rush and Shelden, 2011). Its coaching language and actions recognize what caregivers already know and do to help their child and integrate evidence-based strategies within existing caregiving practices and day-to-day routines that reflect learning needs for the child, parents (or other adults important in the child's life), and the larger family unit. Through family-centered coaching practices, parents actively participate in their child's EI to enhance learning and utilization of existing and new skills.

Despite the Part C value and mandate, real challenges exist to ensuring that family-centered care is implemented at scale or that they are sustainable. First, EIs for children on the autism spectrum often comprise complex, multifaceted packages that require significant training, cost, and resources to learn how to implement directly before coaching others (Glasgow and Emmons, 2007; Trembath et al., 2019). Limited access to training strains this process for programs and providers who do not have easy access to high-quality coaching, consultation, or feedback (Locke et al., 2022). Second, complex interventions require coordination, teamwork, leadership, and a positive climate for successful implementation (Wolk et al., 2020), which has historically been challenging in EI systems (Brookman-Frazee et al., 2020). For example, practitioners need leaders to set expectations, provide resources for engaging parents, and acknowledge performance (Straiton et al., 2023). In addition, the compatibility of the approach to agency values, goals, and current practices matters for the readiness of change by practitioners and organizations, as does the style of engagement between “trainers” and “trainees” (Wainer et al., 2017; Wilson and Landa, 2019; Mirenda et al., 2022). Third, manuals often provide little to no direction for adapting interventions to the context or the individual child and family. Practitioners may be expected to select and combine techniques with limited training or supervision, which may lessen confidence in working directly with parents, and instead, practitioners may revert to child-directed therapy (Campbell and Sawyer, 2007; Fleming et al., 2011).

There is growing efficacy of family-centered care delivered in naturalistic settings, integrating developmental science with applied behavior analysis techniques [i.e., naturalistic developmental behavioral interventions (NDBIs); Schreibman et al., 2015]. NDBIs share core components related to the nature of the learning targets, contexts, and strategies to support the development of early social communication and related skills in children. Research reports positive feedback and experiences for EI providers and parents to engage and teach with NDBIs inside the context of a family's ongoing, daily activities (Hume et al., 2005; Ingersoll and Dvortcsak, 2006; Stahmer et al., 2017; Rogers et al., 2019), as well as routine care (Pickard et al., 2021). The effect remains even when delivered at a somewhat low intensity. This research has been foundational in understanding how to translate NDBIs into systems naturally positioned to serve both children with a known diagnosis and those with an increased likelihood of autism (Vivanti et al., 2018).

The Early Start Denver Model (ESDM; Rogers and Dawson, 2010) is one comprehensive NDBI model that has been validated and replicated in multiple published, randomized trials (e.g., Dawson et al., 2010; Rogers et al., 2019). The ESDM integrates applied behavior analysis principles with sensitive, responsive teaching focused on children's interests, emotional regulation capacity, and a developmental perspective of how skills and behaviors unfold for young children inside their day-to-day routines and the important relationships that make up their young lives (Rogers and Dawson, 2010); two systematic reviews (Waddington et al., 2016; Baril and Humphreys, 2017) and a recent meta-analysis of 12 controlled ESDM studies deemed it a “promising” intervention with significant, positive effects particularly on cognition and language compared with usual care groups, even though most of the studies involved low-intensity (1 h per week) or group services delivered by parents or professionals (Fuller et al., 2020). Multiple studies from its low-intensity parent coaching approach (Rogers et al., 2012), including when delivered remotely, increased parent fidelity without added stress and with concomitant gains in children's language, imitation, and play (Vismara et al., 2009, 2012, 2013, 2016; Estes et al., 2014; Zhou et al., 2018; Gaines et al., 2021; Waddington et al., 2021).

A recent study from Rogers et al. (2022) suggests parent–child gains are possible even when delivery is provided by EI providers learning to coach families in the ESDM. An experimental group of 35 EI providers received webinars and video reviews of their coaching practice with coaches trained to fidelity while the low-income families whom they coached during weekly EI sessions accessed free online parent lessons and materials in the ESDM (see https://helpisinyourhands.org). The control group of EI providers received six webinars on early development and met monthly to review and discuss the materials covered without specific mention of ESDM parent coaching or child interaction strategies. While no significant differences were found between groups in child developmental scores, significant gains with moderate effect sizes did appear for experimental provider fidelity of implementation and in turn for parent delivery compared with the control group. In addition, provider–parent gains averaged from <30 min of weekly coaching across the 6-month period.

Similarly, Mirenda et al. (2021) conducted a large-scale community implementation of a parent coaching training package based on the ESDM with 23 community agencies throughout the Canadian province of British Columbia. While workshop satisfaction and project endorsement were rated uniformly high across EI providers and sites, mean fidelity scores in parent coaching abilities increased for some but not all EI providers across the two time points measured. Providers' job roles and previous clinical experience appeared to account for some of the fidelity discrepancies. Those providers who had strong clinical skills in their roles and were more adept at identifying specific goals, engaging with parents, developing longer term intervention plans, and monitoring progress over time had higher scores than other providers more used to operating within a primarily consultative model that met less regularly with families, engaged in less structured conversations about parent–child progress with target skills, and relied on modeled teaching strategies with the child as the main coaching style with parents. The authors also noted potential coaching variability in response to the wide range of factors surrounding families who participated in the project. The study set no criteria for families apart from having a child younger than 36 months with a diagnosis of autism and the ability to understand and communicate in English. Thus, family demographics and associated strain varied widely with respect to marital status, household income, employment, education, number of other children in the family (either on or not on the autism spectrum), and psychosocial factors (e.g., mental health and history of trauma). Coaching families who have more or less strain may have required providers to adapt their coaching interactions to accommodate specific circumstances, which may have, in turn, contributed to fidelity score variability.

The current study continued this community collaborative research approach, that is, a partnership between researchers and community stakeholders for the provision of ESDM family-centered care through a publicly funded Part C system. The study was divided into three phases. Phase I involved content adaptation of the original ESDM parent-guided manual (Rogers et al., 2012) for contextual fit with Part C delivery, systematic planning between our research-clinical team and a Part C agency, and piloting with six parent–child dyads in a 5-day workshop and six telehealth coaching sessions. Phase II made further refinements to the ESDM parent coaching package from preliminary outcome data, as well as parent and community partner feedback, and implemented a second round of the 5-day workshop and six coaching sessions with four new parent–child dyads. Phase III (the final leg) that is recently complete at the time of this writing supported Part C staff-identified EI practitioners, which can improve implementation progress and sustainment (Damschroder et al., 2009) to use the finalized ESDM parent coaching package with eligible families.

Here, we report the results from Phases I and II. We hypothesized that regardless of the in-person or virtual coaching modality, the abbreviated version of ESDM parent coaching would support critical parent engagement strategies (via parent fidelity, satisfaction, and therapeutic alliance) for increased child learning and social communication skills at the hands of their parents, thus yielding preliminary evidence of the program's feasibility and acceptability before proceeding with community delivery in Phase III. If successful, this “light touch” version of the ESDM may bridge a capacity for families to be served with the intended family-centered care value and mandate of the Part C system in community practice.

## 2. Methods

### 2.1. Setting

As the Community Centered Board (CCB) for Denver, Colorado, Rocky Mountain Human Services (RMHS) is the access point for Denver residents to determine intellectual and developmental disability (I/DD) and developmental delay (DD), receive case management, and access service coordination. Founded in 1992, RMHS is a non-profit organization of ~500 staff who support more than 15,000 Colorado residents through case management and birth-to-adulthood direct service programs. Denver taxpayers dedicate a portion of their property taxes in the form of mill levy funding to benefit Denver residents with an I/DD, DD, or those individuals seeking I/DD eligibility. RMHS contracts with Denver Human Services to administer mill levy funding.

The current study applied each year for Mill Levy funding to carry out all three described phases, one calendar year at a time from 2019 to 2022, in partnership with the Director and Program Representatives of RMHS Mill Levy, Deputy Program Officer of RMHS EI services, and the Associate Director of RMHS Department of Behavioral Health. Video calls were arranged with these key RMHS stakeholders wherein study details were planned and implemented for each phase. A study flyer was provided to RMHS service coordinators and EI providers to share with eligible families, as was a project coordinator made available to provide eligible families from RMHS with more study details for the project phase of the given year. Ethical clearance was obtained from Purdue University, and all clinical research activities adhered to the Health Insurance Portability and Accountability Act (HIPAA) in response to privacy, security, and electronic transaction guidelines.

### 2.2. Participants

Families were eligible for enrollment for either study phase based on the following criteria: (1) at least one parent was the legal, primary caregiver of a child diagnosed with autism or screened and waiting for a developmental evaluation by RMHS; (2) children would not age out of EI during the families' involvement in the study; (3) children received fewer than 10 h per week of EI from RMHS or other intervention sources (e.g., applied behavior analysis); (4) families resided in Denver city and county to benefit from mill levy funding; (5) families were able to consent and complete questionnaires in English; and (6) at least one parent was willing to attend scheduled intervention sessions as part of the study phase they received.

For both study phases, RMHS case managers or EI providers contacted a total of 25 eligible families to provide information about the program and refer those interested to the project coordinator who then consented and enrolled families electronically. Of the 25 contacted, seven families did not respond to the referral; and 10 declined to participate because of scheduling conflicts with work, home demands, or chose to wait until a later evaluation to see whether symptoms continued, thus missing the cutoff age for the study; six parent–child dyads enrolled in Phase I with one dyad dropping out before follow-up measures could be collected because of an emergency situation that demanded their immediate attention. The families enrolled attended a local clinic in the Denver, Colorado, for their baseline and intervention sessions, whereas follow-up coaching was delivered to their homes via telehealth. Four other parent–child dyads enrolled and completed Phase II without attrition. The COVID-19 pandemic forced the 5-day workshop to be delivered to virtual coaching for all contact with families with the exception of one (see Phase II below for further information). Table 1 provides a description of parent–child characteristics at the start of their phased intervention program.

**Table 1:** Baseline parent–child demographics.

	**Phase I**	**Phase II**
	***N*** = **6**	***N*** = **4**
**Child gender**		
Male	5	2
Female	1	1
**Child chronological age (in months)**		
12–24	1	0
25–36	2	3
37–48	3	1
**Child diagnostic category**		
Autism diagnosis	4	2
Autism concern	2	2
**Child ethnicity**		
Hispanic/Latino	2	1
Caucasian	4	2
Multiracial	0	1
**EI intensity (per month)**		
No services at this time	1	0
1–2 h per month	1	2
3–5 h per month	2	2
More than 5 h per month	2	0
**Family status**		
Single	0	1
Married or living with partner	6	2
Separated or divorced	0	1
**Parent education**		
High school/GED	1	2
College degree	2	0
Graduate degree	3	2
**Parent occupation status**		
Not employed outside the home	1	0
Employed part-time	0	1
Employed full-time	5	3
**Household income**		
$25,000–$49,999	1	3
$50,000–$74,999	2	0
$75,000–$99,999	1	0
$100,000–$124,999	2	1

### 2.3. Intervention

The ESDM parent coaching follows principles of adult learning and coaching qualities recognized by Hanft et al. (2004) that empower parents to (a) reflect on what they already know and do to promote learning for their child; (b) practice and evaluate new strategies or opportunities that promote learning; and (c) create ongoing learning for the child when the coach is not present. Its coaching approach emphasizes the importance of collaborative, balanced parent–coach relationships through listening and planning to cultivate decisions and ideas together; the coaches' ability to observe and reflect strategies and skills to parent–child interactions; and coaching with respect, non-judgment, and sensitivity (Rogers et al., 2021). Phases I and II attempted to abbreviate the ESDM from its original parent treatment manual, An Early Start for Your Child with Autism (Rogers et al., 2012), into a coaching curriculum that might lessen implementation barriers for Part C delivery by RMHS EI providers (that study phase is currently underway at the time of this writing). Adaptations were guided by a multidisciplinary group of our partners from RMHS, funding agency representatives, as well as the participating parents and clinical team who provided feedback after review of the parent treatment manual and during Phases I and II. Adaptations addressed parental values shared during and after participation in each study phase; the limited time Part C providers have for learning, planning, and data collection; methods for integrating ESDM parent coaching approaches within the existing Part C Individual Family Support Plan (IFSP); and the limited-service intensity delivered in the community (e.g., as low as 1 h per month). Based on these real-life constraints, adaptations from Phase I to II involved greatly shortening and streamlining intervention materials to fit the study timeline, telehealth format, and workshop style of group and individual coaching. Intervention content prioritized interactive strategies to increase children's attention and motivation for dyadic engagement, non-verbal and verbal communication, imitation, and behavior management; short-term learning goals developed in the study supported families' existing Part C IFSP for continuity; multimodal learning modalities (e.g., text, video, and audio) accommodated parents' learning preferences; and progress monitoring tools and other handouts developed for coaching families identified the teachable moments or other feedback parents provided. Procedural details for each phase follow below.

#### 2.3.1. Phase I

The first phase involved systematic planning between our research-clinical team and a Part C agency for clearly defined roles and responsibilities, content adaptation of the original ESDM parent-guided manual for contextual fit with Part C delivery, and monitoring plans to increase the quality and sustainment of implementation efforts. Adaptations attempted to simplify the ESDM content that Part C providers would share with families into three coaching topics and text-based visual handouts aimed at increasing child social communication skills. Adaptations were then piloted across a five, 2 h per day, in-person workshop with six parent–child dyads and three certified coaches, one of whom was a co-developer of the original ESDM parent coaching content. The workshop took place at a local autism intervention clinic in Denver, Colorado. Day 1 of the workshop introduced parent–child dyads to their coach and completed the ESDM Curriculum Checklist (Rogers and Dawson, 2010) from parent input and observation of parent–child play and other routines that naturally occurred in the time together (e.g., lunch or snack, diapering, or dressing). ESDM Curriculum Checklist items that reflected an observed vulnerability in the child's social communication skills (e.g., not orienting to the parent's voice, imitating a play action or gesture, vocalizing with intention, or following a point to see an object of interest), aligned with the parent's learning priorities in those areas of development for their child, and were feasible for the coach to support within the workshop format and timeline were developed into a minimum of five intervention goals that became the focus of the rest of the workshop and follow-up coaching sessions. Developed goals ranged from increasing children's use of communicative gestures and vocalizations to imitating novel actions in toy play and social games, following parents' requests, and alternating attention between objects and parents to shared enjoyment.

Day 2 of the workshop brought the coaches and parents together as one group while childcare was provided for those without other arrangements. Group discussion and reflection facilitated how parents envisioned using the first two ESDM parent coaching topics, to step into and stay inside their child's spotlight of attention, via following objects and/or play actions that attracted their child's attention for parents to imitate, label, and help to support, elaborate, or add to those interests without taking over, changing, or being excluded from the child's focus; as well as how to set up social spaces to support face-to-face attention, joint interactions, and shared enjoyment. Parents shared feedback, ideas, as well as concerns, about which spotlight strategies they had tried and worked or not worked and what else could be tried or done differently to help them connect, communicate, and foster their child's social communication skills as measured by the intervention goals parents developed with their coach on Day 1 of the workshop.

Coaches actively listened to the values, beliefs, and parenting practices shared and helped to shape parent input into coaching plans that became the focus of parents' direct practice with their child for Days 3 and 4 of the workshops. In each session, coaching was delivered as a collaborative model viewed as a “mutual conversation between two individuals who each have information to share and skills to gain” (Hanft et al., 2004, p. 31). Coaches used the framework developed by Rogers et al. (2021) to support parents as they learned the above-mentioned “spotlight” strategies in the context of engaging their child's attention and motivation in ongoing daily activities in home (e.g., eating lunch, diapering, and putting on socks and shoes) and community settings (e.g., going to the playground and store). Typically, parents engaged in two or more different activities during each coaching session (e.g., singing, reading a book, toy play, and social play) to practice the spotlight strategies and facilitate their child's goals (e.g., teaching the child to point to a picture of interest for the parent to make the silly sound effect, or to copy the parent's clapping gesture when the parent pauses the song in mid-verse). Guided by the coach, who provided feedback and suggestions, parents learned to self-assess each practice activity and reflect on what to keep using or how to make improvements, as needed. Sessions ended with coaches and parents generating practice activities or “action plans” that outlined the spotlight strategies, child goals, and daily activities or routines parents wanted to do with their children. For example, one practice activity was in response to a parent struggling to engage their child who was consistently attracted to touching and staring out of windows. Setting up the social space for the child and parent to have things the child liked and they could do at the window together (e.g., window markers, stickers, pegs that stuck on the window, or soapy water in a spray bottle and a rag) turned a traditionally intense and limiting interest into something interactive and fun for learning to happen.

Day 5 brought back the parents and coaches together as a group for the last day of the workshop with childcare provided again for families in need. In the 1st h, parents shared their experiences with coaching (e.g., what helped or did not help) and observations of their child's behavior in response to the first two “spotlight” strategies. The last hour covered the final ESDM parent coaching topic for parents to turn up their child's spotlight of attention with other play materials, actions, and participatory steps or sequences that would prolong their child's attention and motivation for longer activities and more learning opportunities across intervention goals. Coaches facilitated parent discussions and priorities for how, when, and where they could explore this topic at home across different learning situations and with other family members or caretakers. Reflections resulted in action plans for direct practice with their child that would also serve as the coaching focus for the six bimonthly, 1-h follow-up sessions coaches and parents scheduled before ending the workshop. Those sessions followed the same coaching format in which the coach initially asked about and observed the parent and child progress or challenges with the previous topic before settling on the next topic and goals that the parent and child practiced and that led to an action plan reflective of the parent's feedback, coaching suggestions, and problem-solving the parent planned to use with the child until the next.

#### 2.3.2. Phase II

The second phase delivered the ESDM parent coaching package with the above-mentioned adaptations to four new parent–child dyads. The same 5-day workshop and six follow-up coaching session format described in Phase I was delivered virtually in real-time via HIPAA-compliant video-conferencing software. Parents and coaches connected face to face from their respective locations with a laptop, tablet, or smartphone. Similar to Phase I, families and their coach met online on Day 1 to complete the ESDM Curriculum Checklist and select up to five social communication learning goals for their child that would become the focus of subsequent workshop days and follow-up coaching sessions. Extra time was also set aside if needed to walk through the video-conferencing program features, test the audio–video features, or troubleshoot the internet connections. Coaches and parents then met online as a group without children present for Day 2 of the workshop to discuss and plan how parents would practice the ESDM parent coaching topic to step into and stay inside their child's spotlight of attention. Days 3 and 4 coached individual parents online to use those strategies for teaching their child's social communication goals inside play and other activity moments parents selected at home (e.g., meals, dressing, and bath time). Day 5 ended online as a group with parents and coaches planning and sharing ideas, possible challenges, and questions with the final ESDM parent coaching topic on how to turn up their child's spotlight of attention. That topic served as the focus of the six bimonthly, 1-h online follow-up sessions, which used the same coaching format as described in Phase I.

### 2.4. Implementation

Coaching across both phases was provided by certified therapists in the ESDM. Three of the four therapists were also certified as trainers in the ESDM. In addition, one therapist was a co-developer and author of the original, manualized P-ESDM content and coaching procedures. Coaches' professional disciplines were master's or doctorate-level board-certified behavior analysts or occupational therapists. Sessions with each parent–child dyad were led by one primary coach, and 25% were recorded for separate self and peer-rated intervention fidelity coding across the coaching session structure (i.e., greeting and checking-in; observation and reflection of “warm-up” activity; joint planning and topic introduction; coaching and reflection of each practice activity; closing with a plan, other topics, and goodbyes) and coaching characteristics (i.e., balanced, collaboration; reflective dialogue; and non-judgmental behavior) scored on a scale of 1–4 (1 = not covered; 2 = partially covered; 3 = covered with room for additional coaching opportunities; 4 = fully covered). All coaches maintained expert levels of fidelity (>90%) while working with families.

### 2.5. Measures and data collection

Similar procedures were used for data collection and coding across both project phases. Outcomes were measured at baseline (BL), the end of the 5-day workshop (Post), and the end of the six follow-up sessions (follow-up). Video-based measures (Parent Fidelity, Child Goals, and Abbreviated Curriculum Checklist) were coded from uninterrupted 10-min, parent–child free play interactions across all three time points. For all parent–child interaction videos, parents were instructed to “play with your child as you typically play” and were asked to stay as much on the screen as possible for online recording. No coaching occurred during the data collection videos to observe parents' independent use of the strategies and their effect on children's engagement and learning. Videos were recorded by the coach assigned to the family. All measures were coded by trained assessors naïve to families' intervention experience and time point and who were not involved in coaching sessions. Naïve coders were trained to point-by-point reliability of 0.90 before coding observational data. A second observer coded 20% of observational measures.

Parent-reported questionnaires assessed their perception of parenting efficacy, as well as satisfaction with the ESDM parent coaching content, coaching experience, and overall participation in the program. Families in Phase I completed paper response forms that were mailed in self-addressed, stamped return envelopes, or given copies in-person as needed, whereas electronic forms were provided for parents' online completion in Phase II. Measure collection across phases and project timepoints is reported in Table 2.

**Table 2:** Assessment measures across phases and timepoints.

	**Outcomes**	**Baseline**	**Post**	**Follow-up**
Phase I	Parent	Fidelity	Fidelity Satisfaction	Fidelity
	Child	AbCC		AbCC
Phase II	Parent	Fidelity PSOC FOS	Fidelity	Fidelity PSOC FOS
	Child	AbCCChild goals		AbCCChild goals

AbCC, Abbreviated Curriculum Checklist; PSOC, Parent Sense of Competence.

#### 2.5.1. Parent outcomes

##### 2.5.1.1. Fidelity

Consistent with standard practice in the field (e.g., Stone et al., 2021) and our previous work (e.g., Vismara et al., 2019), video coding was used to measure parent implementation of intervention; 13 intervention skills were rated based on scores of 1 (i.e., no competence) to 5 (i.e., high competence; described in Vismara et al., 2016) with scores of 4 or higher considered fidelity.

##### 2.5.1.2. Parent Sense of Competence

Parents completed the Parent Sense of Competence Scale (PSOC; Johnston and Mash, 1989) at the start and end of the 5-day workshop and after the final follow-up coaching session. The PSOC is a 16-item parent self-report questionnaire designed to measure the degree to which parents feel competent and confident in parenting their child (i.e., efficacy) and the quality of affect associated with parenting (i.e., satisfaction). Items are rated on a 6-point Likert scale with high scores representing high degrees of efficacy and satisfaction. The Efficacy subscale assesses capability, problem-solving ability, and competence, whereas the Satisfaction subscale reflects parenting frustration, anxiety, and motivation. Prior research has shown strong correlations between these subscales and parent–child wellbeing, as well as parenting style (e.g., Rogers and Matthews, 2004) with internal consistency alpha coefficients of 0.76 for the Efficacy subscale and 0.75 for the Satisfaction subscale (Johnston and Mash, 1989).

##### 2.5.1.3. Program satisfaction

Parents completed an in-house 12-item questionnaire at the end of the 5-day workshop that asked to rate their perceptions of the utility of the intervention content, its ease of use, and the quality of coaching relationships across a 5-point Likert scale ranging from 1 (strongly disagree) to 5 (strongly agree). Parents were also asked to describe in an open-ended format the most and least helpful aspects of the program.

##### 2.5.1.4. Family outcomes survey

Parent completed the FOS at baseline and follow-up. This survey was developed as a measure for evaluating the effectiveness of early intervention programs for children with disabilities (Bailey et al., 2011). It consists of a total of 41 items across two subscales: Family Outcomes and Helpfulness of Early Intervention.

#### 2.5.2. Child outcomes

##### 2.5.2.1. Child goals

Achievement of child goals was measured by the number of intervention goals parents attempted and successfully taught from their child's list vs. missed opportunities that could have been practiced or attempted and not followed through (see similar procedures described in Vismara et al., 2019).

##### 2.5.2.2. Abbreviated curriculum checklist

Child change was measured by the number of proximal skills that coders observed from an abbreviated version of the ESDM Curriculum Checklist, a criterion-based measure of child development. The abbreviated version highlighted 37 of the 480 original items organized in eight developmental domains affected by autism in early development: receptive understanding of gestures and words, expressive use of gestures and words, joint attention, social interaction with adults, imitation, cognition, and play skills (both functional and symbolic), and behavior management (e.g., remains in the activity or sits willingly to participate). Each item was rated as “acquired” in which children spontaneously emitted the skill without prompting from their parents; “partial/prompted” in response to parental verbal, gestural, and/or physical scaffolding; or “unable/unwilling” even with parent scaffolding. This tool and probed items, rather than a standardized developmental assessment measure, were selected as a closer proxy to the actual skills that children were being taught by their parents through P-ESDM coaching. Items are summed to create a total score for analysis.

## 3. Results

### 3.1. Data analysis

All parent–child data including the dyad in Phase I who withdrew prematurely from the study were included for data analysis. Variables collected within a single-subject design framework (Parent Fidelity and Child Goals) were first analyzed through visual analysis. These variables were then analyzed using Tau-U, a non-parametric rank correlation effects size, with an online calculator (ktarlow.com/stats/tau; Tarlow, 2016). The remaining variables were analyzed with paired-samples *t*-tests.

### 3.2. Phase I

#### 3.2.1. Parent fidelity

**Figure 1:**
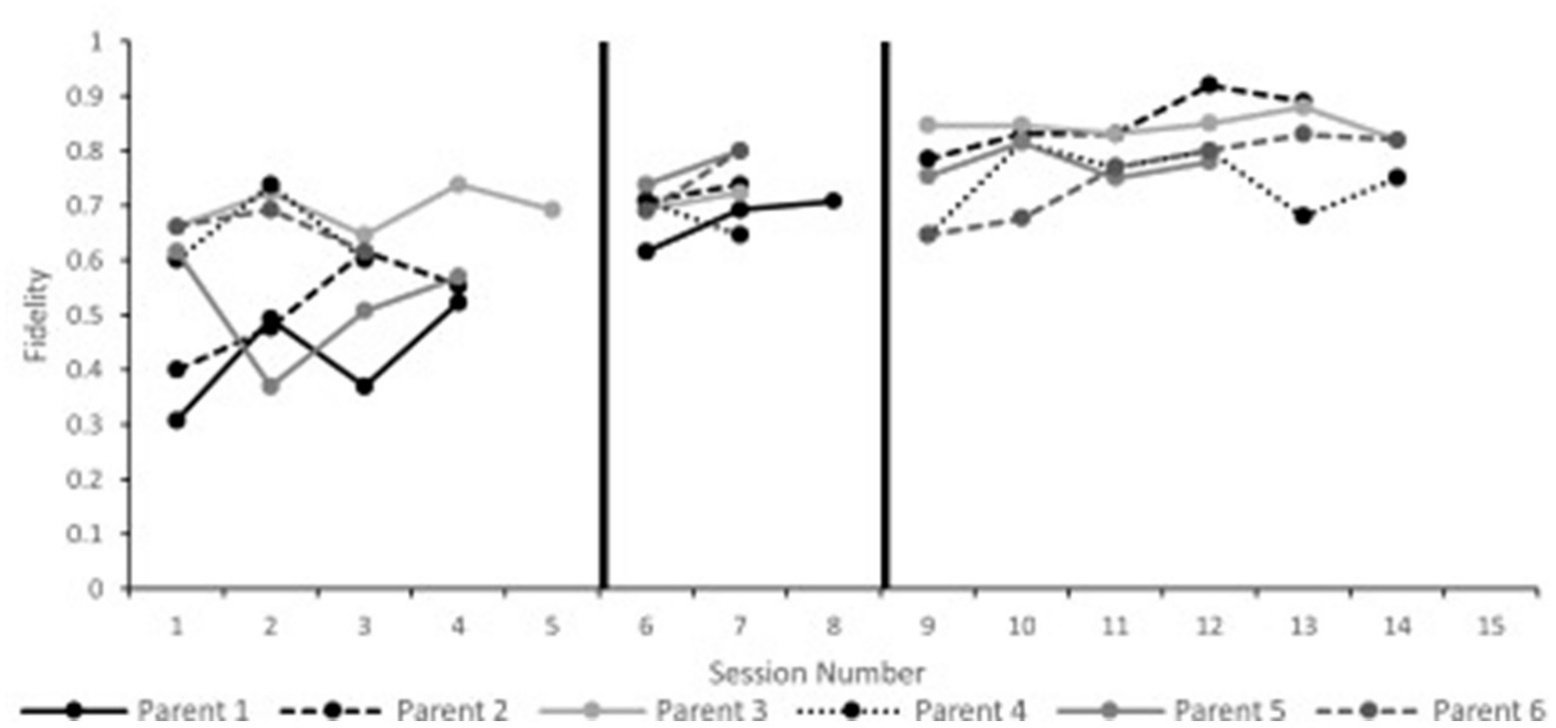
Phase I parent fidelity.

Parent fidelity scores are presented in Figure 1. Visual analysis of the data indicates that all parents improved across the course of the study. Of the five parents who remained in the program, four demonstrated significant changes in their fidelity scores (Parents 2, 3, and 5). Although parent six did not have a significant effect size, they reached fidelity on their last three probes. Tau effect sizes are reported in Table 3.

**Table 3:** Tau effect sizes for parent P-ESDM fidelity for Phase I.

**ID**	**Tau**	**SE**	***P***
1	0.76	0.35	0.052
2	0.72	0.30	0.011
3	0.58	0.32	0.023
4	0.45	0.381	0.124
5	0.73	0.31	0.014
6	0.48	0.38	0.101

#### 3.2.2. Abbreviated curriculum checklist

Children demonstrated significant improvement in scores on the AbCC [*t*_(4)_ = 8.75, *p* = 0.001]. At post-test scores ranged from 14 to 40 on the AbCC compared with a range of 3–30 at the start of intervention. Family 6 did not complete the time 2 AbCC and was therefore not included in analyses.

#### 3.2.3. Parent satisfaction

A total of nine parents completed the post-workshop survey. All six primary participants and two second caregivers. All items were highly rated with average scores ranging from 4.44 to 5.00. The lowest ranked item was related to the length of the workshop. Analysis of the comments in response to a prompt asking for improvements revealed that parents wanted more time with coaches and more opportunities to practice. One parent suggested, “If it was just 1–2 days longer for more practice.” Additional comments on the survey emphasized the impact of receiving individualized feedback from knowledgeable therapists. One parent wrote for a strength of the workshop, “time with [therapist] and individual coaching specifically identifying [my child's] specific subtle cues at his developmental level.” Parents also highlighted the importance of connecting with other parents. As one parent wrote, “I love talking with other parents.” Overall quantitative and qualitative feedback on the survey indicated high parent satisfaction with the program.

### 3.3. Phase II

#### 3.3.1. Parent fidelity

**Figure 2:**
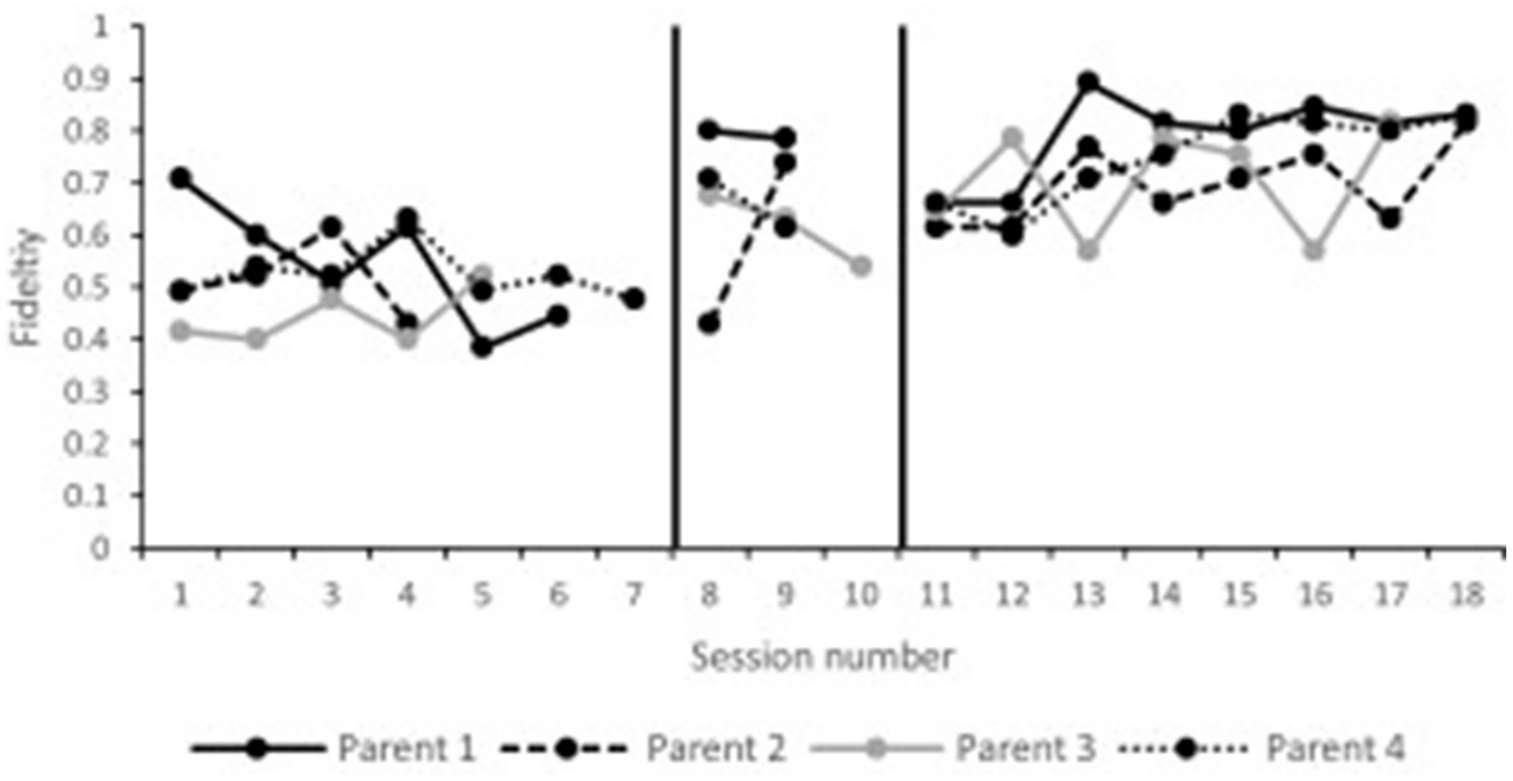
Phase II parent fidelity.

Parent fidelity ratings across the timeline of phase II are presented in Figure 2. All parents were consistently below fidelity during the baseline probes. During follow-up, all parents had at least one probe at or above fidelity. Tau effect sizes reported in Table 4 confirmed visual analysis of the data. All parents significantly improved their implementation of the intervention from baseline to follow-up sessions.

**Table 4:** Tau effect sizes for Phase II parent fidelity.

**ID**	**Tau**	**SE**	***P***
1	0.76	0.277	0.008
2	0.656	0.322	0.033
3	0.712	0.3	0.015
4	0.678	0.3	0.014

#### 3.3.2. Parenting sense of competence scale

Parents reported an increase in self-esteem from pre-intervention (range 65–78) to follow-up (range 83–97), *t*_(3)_ = 7.91, *p* = 0.004.

#### 3.3.3. Family outcomes survey

Parents reported an increase on the Family Outcomes subscale of the FOS from pre-intervention (range 2.63–4.25) to follow-up (range 4.42–5.00), *t*_(3)_ = 5.35, *p* = 0.01. Parents did not report an increase on the Helpfulness of Early Intervention subscale from pre-intervention (range 3.65–5.00) to post-intervention (range 4.82–5.00), *t*_(3)_ = 1.96, *p* = 0.15. All parents had a mean score above 4.00 at post-intervention, indicating a satisfactory outcome (Ueda et al., 2015).

#### 3.3.4. Abbreviated curriculum checklist

Children made significant progress on the total score of the AbCC from pre-intervention (range 5–21) to follow-up (range 13–31), *t*_(3)_ = 3.97, *p* = 0.03.

#### 3.3.5. Child goals

**Figure 3:**
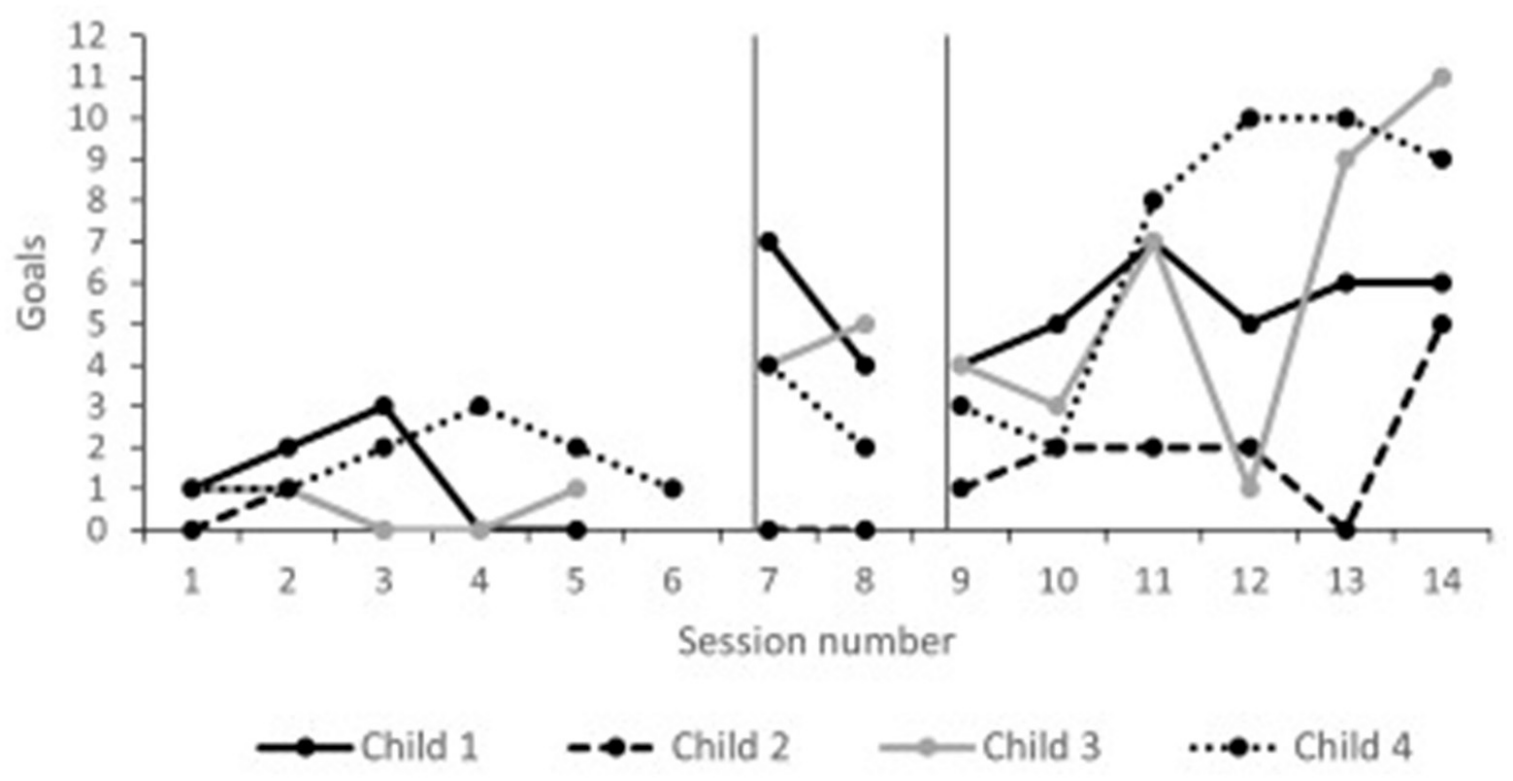
Phase II child goals.

Child goals across the timeline of Phase II are presented in Figure 3. Children were meeting a few steps within their goals during the baseline period (range 0–3). During follow-up, all children were regularly meeting multiple goals (range 0–11). Tau effect sizes reported in Table 5 confirmed visual analysis of the data. All children significantly increased the number of met goals from baseline to follow-up.

**Table 5:** Tau effect sizes for Phase II child goals.

**ID**	**Tau**	**SE**	***P***
1	0.76	0.277	0.008
2	0.656	0.322	0.033
3	0.712	0.3	0.015
4	0.678	0.3	0.014

## 4. Discussion

In spite of parent coaching considered a best practice in working with young autistic children, it is infrequently delivered in community settings (Brookman-Frazee et al., 2010; Dingfelder and Mandell, 2011; Straiton et al., 2023). One factor that may contribute to this research–practice gap is the design of methodologies with limited to no perspective or input from practitioners, autistic individuals, and their families (Guldberg, 2016; Carruthers et al., 2022). This study attempted to draw on those experiences through a research–community partnership (Brookman-Frazee et al., 2012) with a Part C agency and participating families. We report initial acceptability, feasibility, and parent–child outcomes on an adapted, abbreviated version of the original ESDM parent coaching manual that was piloted in-person and virtually with two sets of families before supporting EI clinicians in the Part C system to coach families with the materials.

Parent-mediated interventions involve changes to the ways parents may interact with their children (Stahmer et al., 2017; Rogers et al., 2022). Mediation studies of two intervention trials found that the magnitude of change in parental behavior was the key driver of improvement in child outcomes (Pickles et al., 2015; Gulsrud et al., 2016; Shih et al., 2021). Parents who developed the intervention strategies and understood how to adjust their actions in relation to their child facilitated larger gains in their child's outcomes (Pickles et al., 2015; Shire et al., 2016). Therefore, parent learning and fidelity matter.

To this first objective, we found that parents' ability to use the ESDM increased over time with higher scores reflecting interactions closer to ESDM principles. Fidelity overall averaged 79% and fell just below the targeted implementation threshold of 80%. An important step in supporting adult learning and behavior change is to identify facilitators and barriers that individuals experience in their efforts to implement that behavior. In parent-mediated autism interventions, parents have previously highlighted certain aspects that “make or break” their ability to use the intervention itself, such as strategies that can be used in everyday situations (McConnell et al., 2015; Pickard et al., 2016), the strength of their relationship with therapists (Johnson and Hastings, 2002; Pickard et al., 2016), and feedback on progress (Raulston et al., 2018).

How easy new behaviors are to learn and use, as well as their adaptability across different situations, is important for change to happen and sustain. In our two samples, parents felt very positive about the strategies. They reported the ease and regularity they could use strategies in daily routines during a typical week. Parents highlighted personal factors that changed as a result of learning to deliver the strategies themselves with their child, such as their level of confidence in their parenting skills, their knowledge of autism, and their relationship with their child, as well as with their wider family. Also reflected in parent's comments is the alignment between their values and family lifestyle with the intervention that may have contributed to their adherence. Supportive alliances with therapist, confidence and compatibility with the therapy, and belief in their own capabilities reiterate facilitators of change viewed by parents in previous studies (Johnson and Hastings, 2002; Solish and Perry, 2008; Moore and Symons, 2011; Pickard et al., 2016; Carruthers et al., 2022).

In addition, parents reported benefits from being introduced to one another during the workshop, as well as the encouragement they received from the group dynamics of listening and sharing experiences. Working out how to “balance” the therapy alongside other demands on their time or how to adapt the strategies to accommodate a child's changeable behavior may be common challenges for parents undergoing the intervention. Sharing knowledge, acknowledging hardships, and offering encouragement to each other may influence behavior change and add therapeutic benefit (Borek et al., 2019; Biggs et al., 2020; Robinson and Weiss, 2020). Some previous work lends support for this (Stahmer and Gist, 2001).

While all parents continued to use the strategies in some way, it is also important to recognize that some parents could be better supported in achieving the desired level of implementation. An important component in the context of parent-mediated interventions is the extent to which parents are willing, ready, and able to take on this role with their child. In this study, all parents were motivated to enroll and agreed with the benefits of such an approach. However, some parents could still feel uncertain about a parent-mediated approach and need time to see its full value. Alternatively, parents may see the potential value but not feel equipped with the requisite skills and knowledge, which in turn leads to uncertainty with whether they will acquire them. Child behavior, mood, and needs may also limit or interfere with opportunities to use the strategies. Being able to tailor the intervention (e.g., include the child's interests or recognize and respond to a child's attempts) and adapt them across different situations and needs are aspects of the ESDM aimed at addressing these kinds of barriers; however, such issues can still remain a challenge for many parents. Different intervention approaches will suit parents to varying degrees, including over time, and as children age and their needs shift, parenting styles may also adapt. The compatibility of whether a parent-mediated intervention is what parents want, as well as the “readiness” of parents and/or timeliness of the intervention, are interesting considerations for implementation.

Another possible barrier to parent implementation is how planned vs. spontaneous parents become in when and how to use intervention strategies. Parents may forget to use them the less automatic and more they have to consciously think about it. Although this study cannot underpin these differences, it is possible that differing parenting styles at baseline and the opportunities they afford or miss may play a role. In addition, adult learning naturally benefits from varying styles of coaching techniques (Friedman et al., 2012), particularly as parent-mediated interventions can be complex and require extensive time and expertise to achieve fidelity (Rogers et al., 2012). Not many studies have explored how parents' capabilities, characteristics, and contexts may interact with their use of intervention strategies but those done indicate the importance of self-efficacy, understanding of child development (Siller et al., 2014), capacity for reflection and self-evaluation (Siller et al., 2018), and parental stress (Estes et al., 2014; Stadnick et al., 2015). As the field seeks to understand whom and under what conditions interventions are most effective, it seems crucial to consider how parental characteristics may influence the learning and use of techniques (Trembath et al., 2019).

The progress parents reportedly observed in their child and that they attributed, at least in part, to the ESDM could also influence the amount of learning they initiated with children. Video-coded measures by a naïve evaluator showed parents actively involving their child through developed learning objectives (e.g., imitating an action with a toy; pointing to reference a named picture or object; verbalizing three object or action words in context) in addition to other ancillary skills noted on the ESDM Curriculum Checklist that were not the focus of coaching sessions. Such change acts as reinforcement and sustains motivation to continue using the intervention (Stahmer et al., 2017). Although not standardized change, the proximal development for both parent–child learning is consistent with recent evidence that parents' improved sense of competence mediates the relationship between their behavior and their improved understanding of their child (Brookman-Frazee et al., 2020).

With the exception of one parent who asked that coaching switch from virtual to in-person, subtle differences appeared between the two coaching modalities. Families were equally likely to attend sessions. They showed similar capacity to use the interactive strategies in their most natural environment (i.e., the home) and reported similarly positive responses to the program. Virtual coaching was mentioned by several parents as a powerful learning technique because of the number and range of real-life situations that could be observed and supported with the intervention strategies. However, certain behaviors or needs (e.g., sleep, toileting, and eating) may be inappropriate or more challenging to coach at a distance. As was the case with one parent in our study, she found her child's frustrating and resulting behaviors difficult to manage when coached virtually during the individual sessions of the workshop. During periods of the child's upset from a preferred activity having to end or her trying to take a turn, the mother could not split her attention between the child and coach or always hear the coach's voice and feedback. Instead, she found that her attention and concentration were better suited to in-person coaching and in turn felt more capable of parenting her child.

The abbreviated ESDM parent coaching program offered in two different modalities attracted families from different ethnicities, education and employment backgrounds, and income levels. Almost half of the group self-identified as People of Color, and almost three-quarters reported below the real median household income (census.gov). Approximately one-third did not complete schooling beyond high school, all but one employed, and nearly one-quarter were separated or single parents. The diversity within this program demonstrates the inclusivity of this model for people from historically marginalized communities to participate at least in a short-term program and to learn and improve in their delivery of the intervention toward fidelity. As child change in parent-mediated models is dependent upon the parents' ability to deliver the intervention, and as parent delivery is dependent upon how and what they are coached, the results are encouraging that both of these links of the chain are positively affected by the implementation model being tested here. Initial findings also underscore the feasibility of the model, across a range of families, and its potential to be embedded into community pathways.

A potential criticism of coaching families concerns intensity of intervention and the developmental gains made or missed out on from the number of hours per week children receive (Rogers et al., 2012, 2022). Particularly when there are few intervention hours offered to children, professionals can help families to maximize intervention hours, first by coaching to help them provide high-quality, high-frequency actively engaged learning at home and other meaningful environments; and second by steering them to high-quality interventions available in the community. Coaching does not have to replace or act as a substitute for intensive services. But it can offer a lifeline and initiate a support system for an “act now” mentality (Rogers et al., 2012). The use of implementation science frameworks within the autism field is increasing (Brookman-Frazee et al., 2020), but there are very few examples of using these frameworks with parents (Rieth et al., 2018; Carruthers et al., 2022). Our study contributes to this focus with personal characteristics (e.g., skills, goals, and intentions), which have not been widely studied in the context of parent-mediated autism interventions (Siller et al., 2018; Trembath et al., 2019).

The current study adds to the growing literature attesting to the feasibility of implementing evidence-supported interventions within community settings using a community-partnered participatory approach. Families participating in this research were recruited directly from the Part C system in their local community and came with a diverse set of backgrounds and learning needs. The study used quantitative measures of parent fidelity with outcomes that suggested a high level of intent and perceived favorability to continue implementing the intervention after the study ended. Although coaching was abbreviated, a short timeline for participation may not have diminished parents' memory of the experience and thus impacted their responses to the questionnaires. Their first-hand insights into the ESDM highlight what helps and does not help support their use of the strategies at home.

However, several limitations should be acknowledged. Although consistent with the scope of a pilot feasibility study, the small size of our sample, coupled with the variability of parent–child outcomes, limits any conclusions about effectiveness that can be drawn from the study. Nor can a small sample size evaluate the extent to which factors such as socioeconomic status, culture, or child's age influence parent learning and fidelity, which would have provided a more in-depth contextualization of the findings. The lockdown during the COVID-19 pandemic also made for complications with recruitment, the randomized controlled trial design, and longer follow-up period initially planned for Phase II. It is possible that parents and children may have shown stronger responses over a longer period of time; however, long-term maintenance is an important focus. To maximally benefit families from parent-mediated interventions, we need insight into the extent of sustained use of the therapy, or how it evolves over time. Another limitation was the lack of systematic questioning during the COVID-19 lockdown to ask parents about the potential impact of those circumstances. Finally, children's proximal outcomes tapped into progress toward individual goals and should be complemented with broad outcome measures of cognitive and adaptive functioning in future research. This will help the field better understand the mechanisms underpinning longer term outcomes.

## 5. Conclusion

In conclusion, this study applied a community-partnered participatory approach, which gave us the unique opportunity to directly incorporate feedback and insight from our community partners and families ultimately intended to be the end-users of the intervention. In doing so, we were able to adapt our intervention content, as well as coaching characteristics and process, in a way that facilitated implementation of the ESDM within the context of families' homes and during a portion of the COVID-19 pandemic. Furthermore, this collaboration provided us with insight into potential facilitators (i.e., what supports or works well) and barriers (i.e., what limits or does not work) to incorporating a family-centered model into the Part C system and of the training and implementation by Part C providers in the next phase of our study. This is an important step toward successful widespread dissemination of early intervention in the community with preliminary feasibility, acceptability, and benefit for children and their families.

## Data availability statement

The raw data supporting the conclusions of this article will be made available by the authors, without undue reservation.

## Ethics statement

The studies involving human participants were reviewed and approved by Purdue University. Written informed consent from the participants' legal guardian/next of kin was not required to participate in this study in accordance with the national legislation and the institutional requirements.

## Author contributions

LV and LN collected and managed data with collaboration from staff in the community. CM created and executed the analytic plan. All authors contributed to the drafting of the manuscript. All authors contributed to the article and approved the submitted version.
